# Successful Surgical Management of an Invasive Adenocarcinoma Within a Duplicated Gallbladder by an Extensive Hepatic Resection: A Case Report

**DOI:** 10.7759/cureus.34440

**Published:** 2023-01-31

**Authors:** Zaid Mesmar, Ahmad Alqassieh, Ammar O Mahmood, Jared White

**Affiliations:** 1 General Surgery and Urology, Jordan University of Science and Technology, King Abdullah University Hospital, Irbid, JOR; 2 Transplant Surgery, Medical University of South Carolina, Health University Medical Center, South Carolina, USA

**Keywords:** hepatobiliary surgeries, adenocarcinoma, duplicated gallbladder, gallbladder anomalies, gallbladder

## Abstract

Identifying a duplicated gallbladder is a rather rare entity, but a well-described phenomenon within the current literature. Although this finding has been described in numerous case reports, management remains poorly defined and the diagnosis is often difficult. We present a case of a patient with a suspected duplicated gallbladder versus a choledochocele that was diagnosed later on with adenocarcinoma within a duplicated gallbladder during surgical management requiring extended hepatic resection for curative intent. This case emphasizes the importance of radiological techniques in diagnosing such rare cases and the surgical approach of managing adenocarcinoma in the presence of this rare anatomical malformation.

## Introduction

Duplication of the gallbladder has an incidence of approximately 1:4000 [1]. However, the exact incidence of this rare gallbladder anomaly cannot be accurately assessed, since the only cases which can be identified are those that became symptomatic or were encountered as incidental findings during surgeries, imaging studies or at autopsy [1].

Gallbladder duplication can present as a clinical challenge due to difficulties in the diagnosis and identification of contributory symptoms in addition to the most appropriate surgical management. Recognizing this anomaly and its various types is mandatory as it can complicate gallbladder disease or an otherwise routine biliary surgical procedure [2]. These patients can present as asymptomatic duplicated gallbladders or with clinical symptoms such as cholecystitis, cholangitis, and rarely but more importantly, adenocarcinoma. This case report highlights the diagnosis and management of a suspicious mass identified within a duplicated gallbladder that was proven to be adenocarcinoma requiring a hepatic resection intraoperatively. To our knowledge, only six reported cases exist describing such a presentation in the literature with relative paucity of expert opinion on approaches to medical and surgical management [3-9].

## Case presentation

**Figure 1:**
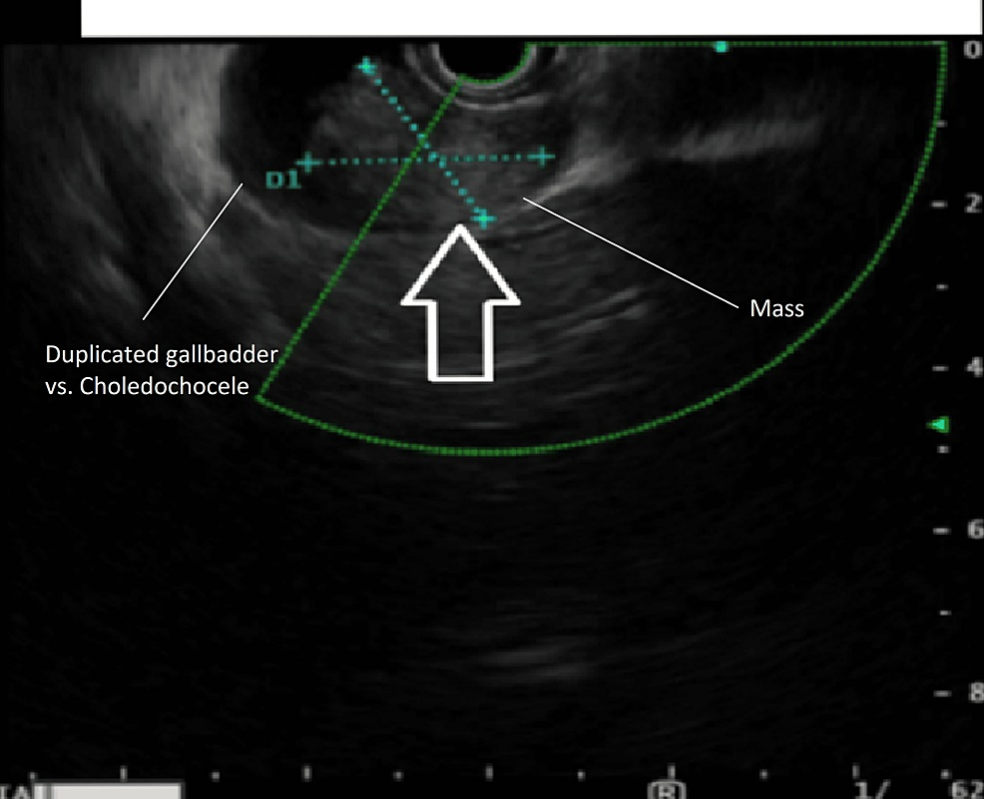
EUS with an arrow pointing to an ovoid mass that appears to be arising from a choledochocele versus duplicated gallbladder. EUS, endoscopic ultrasound

**Figure 2:**
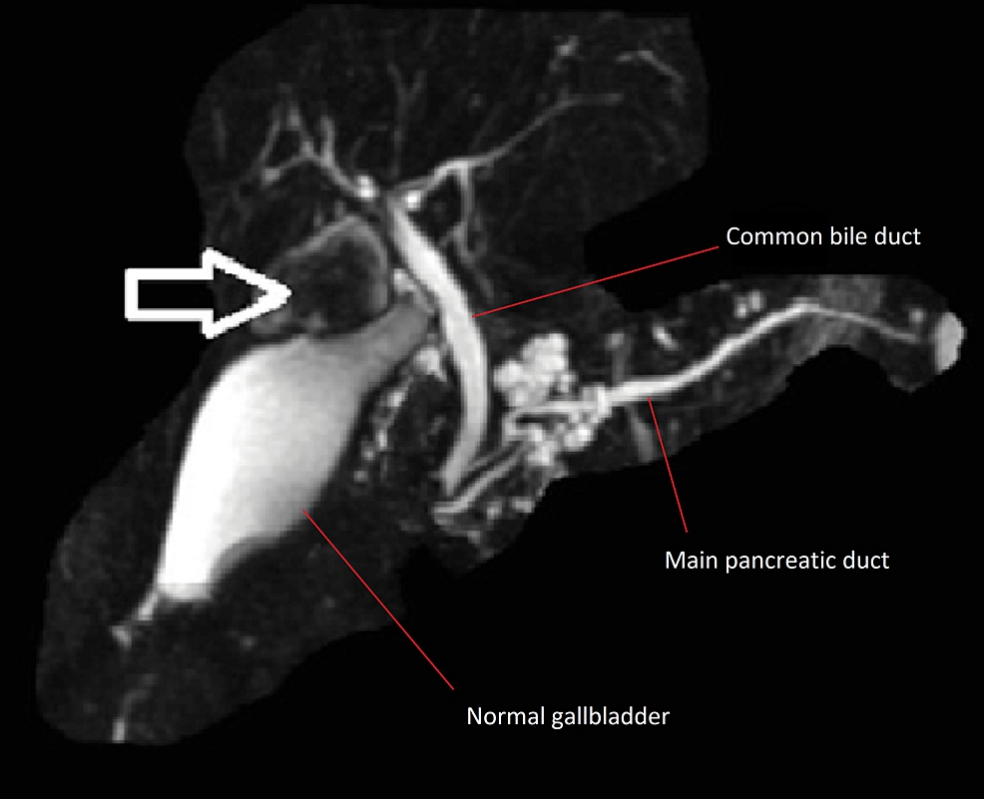
MRCP with an arrow pointing to the duplicated gallbladder emanating from the proximal right intrahepatic duct takeoff with a filling defect concerning for a mass. MRCP, magnetic resonance cholangiopancreatography

The patient is a 70-year-old Caucasian female who presented with complaints of intermittent epigastric pain for three months prior to presentation for which an ultrasound from an outside hospital reported the presence of a solid-appearing mass located within a cystic appearing lesion embedded in the gallbladder bed and measuring 2.4 cm x 1.8 cm x 2.6 cm raising concern for cholangiocarcinoma (images of the study could not be obtained). An endoscopic ultrasound (EUS) was scheduled for the patient showing the presence of an ovoid mass that appeared to be arising from a choledochocele versus duplicated gallbladder, and no regional lymph nodes were observed (Figure 1). Before that, a chest CT scan was done and a MRI, metastasis has been ruled out, as the clinical staging was decided as cT1N0M0. There were also small cysts scattered throughout the head of the pancreas, with a size less than 1 cm each. A magnetic resonance cholangiopancreatography (MRCP) showed a duplicated gallbladder emanating from the proximal right intrahepatic duct takeoff with amorphous filling defect concerning for soft tissue mass with otherwise normal biliary tree (Figure 2), and no regional lymph nodes. The patient’s past medical history was otherwise unremarkable with a normal cardiopulmonary assessment and no risk factors for cholangiocarcinoma. Of note, the patient’s carbohydrate antigen 19-9 (CA19-9) was within normal limits at 8.0 u/mL (ref normal <37 u/mL). Complete blood count (CBC), carcinoembryonic antigen (CEA), lactate dehydrogenase (LDH), and liver function test (LFT) measurements were all within normal range.

Given the patients’ lack of underlying liver disease and adenopathy on MRCP, and the favorable surgical location, we prepared to proceed with an open cholecystectomy to include the duplicated gallbladder with the potential for partial hepatectomy and possible biliary reconstruction in the event of intraoperative frozen sections identifying invasive carcinoma.

**Figure 3:**
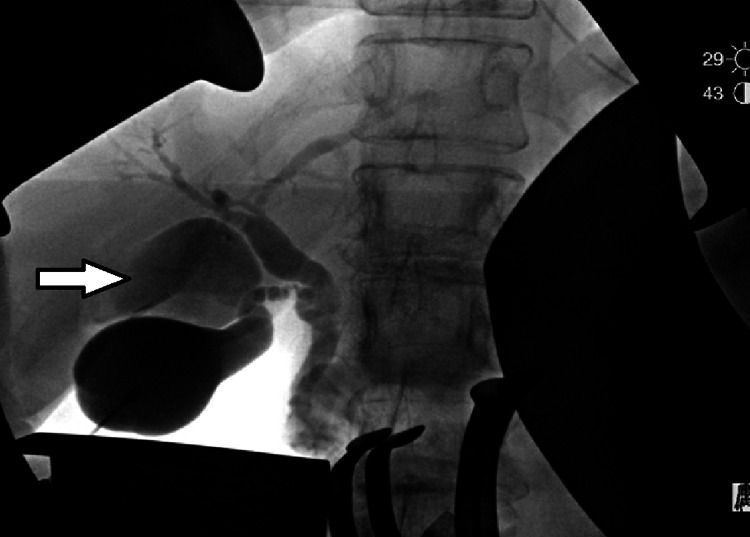
Intraoperative cholangiogram with an arrow pointing to the duplicated gallbladder.

Intraoperatively, a low central venous pressure management (<10 mmHg) with minimal IV fluids was prepared using central venous access and radial arterial line for invasive monitoring. We began with a midline subxiphoid incision with safe entry into the abdomen. We palpated the peritoneum and intrabdominal viscera to ensure no obvious evidence of carcinomatosis. Finding none, we performed a right subcostal extension of our incision for appropriate exposure and placed retractors accordingly. The falciform ligament was divided and the triangular ligaments were cauterized to fully mobilize the liver. We performed a complete portal dissection and skeletonized the vasculature removing all periportal lymph nodes and lymphatic tissue, clearly identifying the common bile duct, portal vein, and hepatic arteries. The visible gallbladder appeared grossly normal, and we used a fundus-down approach to remove the gallbladder from the fossa to the level of the cystic duct. At this point, we could clearly see the duplicated gallbladder at the base of the cystic duct. We elected to place a cholangiogram catheter into the gallbladder and performed an on-table cholangiogram with Omnipaque contrast and fluoroscopy. The duplicated gallbladder did opacify signifying a direct connection to the biliary tree consistent with the expected duplicated gallbladder with intra cholecystic opacity consistent with the presumed tumor (Figure 3). We carefully divided the cystic duct in addition to the ductal component of the duplicated gallbladder en-bloc marking the proximal duct with clips in the event of malignancy on margin assessment. The specimen was marked for the hepatic side of the two gallbladder structures and taken to pathology for further assessment.

**Figure 4:**
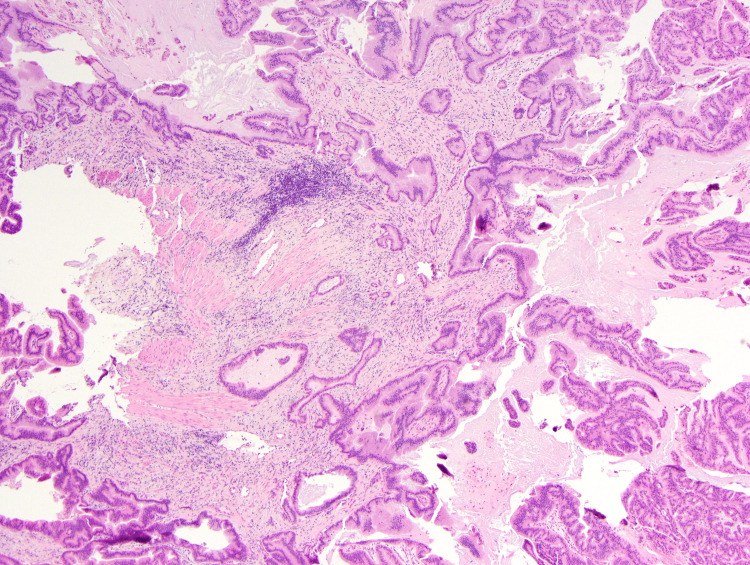
Histological staining with hematoxylin and eosin (H&E) at 40x of the intraoperative frozen section concerning for invasive carcinoma.

The gallbladder and duplicated gallbladder were incised showing a pedunculated and friable soft tissue lesion approximately 2 cm within the duplicated gallbladder that appeared consistent with invasive carcinoma microscopically (Figure 4). Given the location of the mass embedded in the gallbladder bed and adhesive to the liver tissue, the size of the lesion and the invasive carcinoma suggested by the microscopic frozen examination, we elected to perform a right hepatic 4A sparing trisegmentectomy in order to minimize the potential for positive margins or recurrent disease in the future. The right hepatic artery was identified and divided. The right portal vein was encircled and stapled with the Echelon™ stapler (Johnson and Johnson, New Brunswick, NJ) white load. The liver demarcated expectantly at Cantlie’s line, was scored to include segment 4B, sparing 4A. The right lobe was mobilized from the vena cava, ligating numerous venous branches to the vena cava with ligatures and clips up to the level of the right and middle hepatic veins. The right hepatic vein was encircled in addition to the middle hepatic vein for vascular control. We then used a combination of ultrasonic dissection and high-energy devices with clips, suture ligatures, and Echelon™ stapler blue loads to transect the liver. The right hepatic duct was identified, sharply divided, and the remnant stump over sewn with a margin assessment that was negative for malignancy. Finally, the right hepatic vein was stapled with an Echelon white load, and the specimen was removed from the field. Hemostasis was excellent, blood loss minimal requiring no blood products for transfusion. A Jackson-Pratt (JP) drain was placed in the right upper quadrant and the abdomen was closed. Frozen examination of all critical surgical margins was negative.

Postoperatively, the patient was transferred to a general floor bed with expectant management of pain control, IV fluid with electrolyte replacement, and advancement of diet with return of bowel function. The JP drain was serosanguinous with no evidence of bile leak, and was removed on postoperative day 4. The patient was discharged on postoperative day 5 following an unremarkable recovery. Peak total bilirubin was 1.8 mg/dL on postoperative day 1 and decreased to 1.2 mg/dL by discharge. Transaminases decreased to within normal limits by postoperative day 3. At the two-week post-op follow-up all of the patient’s labs had normalized and she was doing extremely well without any complications.

**Figure 5:**
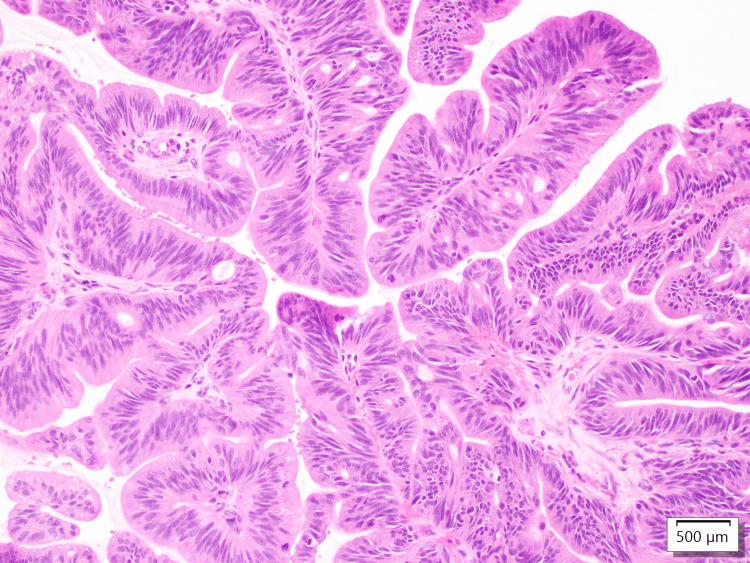
Histological staining with hematoxylin and eosin (H&E) at 200x showing ICPN with high grade intraepithelial neoplasia. ICPN, intracholecystic papillary neoplasm

**Figure 6:**
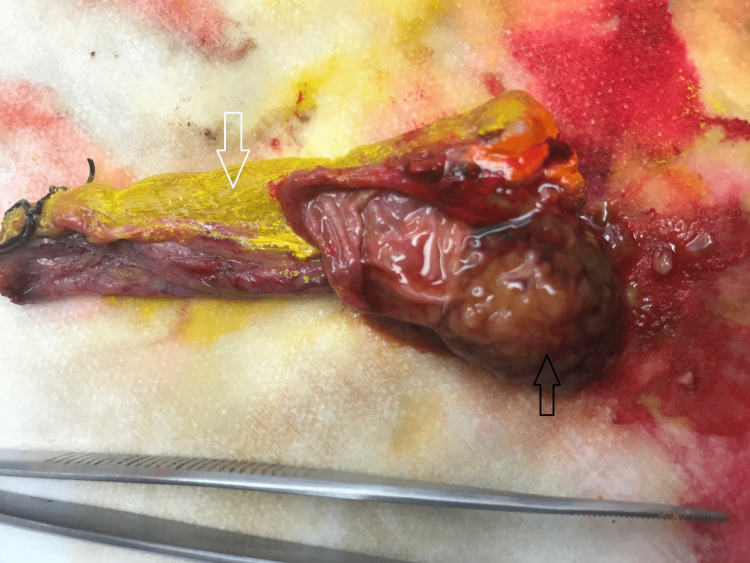
Intraoperative image showing the duplicated gallbladder with black arrow pointing to the tumor in the posterior gallbladder and white arrow to the anterior normal gallbladder.

From a final pathological standpoint, the tumor size was 2 cm by gross measurement and histopathology showed focal adenocarcinoma arising in the intra-cholecystic papillary neoplasm (ICPN) with high grade intraepithelial neoplasia (Figure 5). Histologic grade was well-differentiated adenocarcinoma (g1) and the tumor site was the body of the posterior duplicated gallbladder (Figure 6). The postoperative pathological staging was reported as pT1N0M0. The hepatic specimen showed no evidence of biliary dysplasia and benign vascular and parenchymal margins. Lymph nodes were negative for metastasis with no perineural or perilymphatic invasion.

An oncology referral was completed regarding any future risks of carcinoma from the small focus found in her duplicated gallbladder and lack of disease in the right hepatic lobectomy specimen, but the favorable pathology with negative lymph nodes and negative tumor margins in the liver specimen warranted no further chemotherapy or radiation therapy per final recommendations. The patient has been followed for the past 2.5 years and is doing well and free of disease by a 6-month MRI, and a 18-month CT abdomen and pelvis with contrast.

## Discussion

Gallbladder duplication is a rare congenital anomaly with the exact incidence being unknown, but reported to occur approximately 1 in 4000 births [10]. The exact pathophysiology of this disease remains unknown. Several proposed theories exist, as described by Boyden suggests numerous accessory vesicles present during the fifth to sixth week of embryogenesis which regress, with lack of regression leading to a duplicate system [11]. Other theories include splitting of the cystic primordium and accessory cystic primordium [12]. Classification systems exist are based on embryological origin. The most well-accepted classification described by Harlaftis categorizes duplication as Type I, split primordium or Type II, accessory gallbladder [12].

There is no current literature to support increased incidence of biliary disease due to this anomaly [13]. A duplicated gallbladder can often appear similarly to a Todani II bile duct cyst on imaging, which is known to have an increased risk of malignancy [14]. Malignancy within duplicated gallbladders has been described in several case reports [3-9]. There are currently no formal guidelines for management of this extremely rare presentation. The most prognostic factor is the depth of tumor invasion (T stage). So, pathologic examination of all gallbladder specimens removed for benign disease is necessary to detect gallbladder carcinoma at an early stage [15]. In order to provide the best oncological outcome for the patient to prevent any possibility of cancer being left behind and to ensure negative margins. As the frozen sections are not always diagnostic and oftentimes 'negative' margins can back as positive on permanent stains, we elected to be more aggressive to ensure the best oncological resection in this scenario, by performing a trisegmentectomy of the liver.

Accurate preoperative diagnosis of such anomaly is of great importance in order to prevent postoperative complications. EUS and MRCP are considered ideal diagnostic modalities for preoperative detection and characterization of the anomaly, but differentiating between choledochal cysts vs duplicated gallbladder remains challenging using such techniques [16]. Nevertheless, MRCP demonstrated the presence of the duplicated gallbladder and visualized the mass within the gallbladder. This helped us assess the biliary system and decide the proper surgical management. Intraoperative cholangiogram was also helpful in confirming the direct connection and its site of the duplicated gallbladder with the biliary system illustrating the importance of the image in managing this rare type of gallbladder anomaly. We have treated the patient with an extensive hepatic resection following the standard of care guidelines, and because the diagnosis was unclear, an intraoperative frozen section confirmed cancer, which supported our decision in definitive resection. Surgeons should be aware of the possibility of malignancy as management can range from simple cholecystectomy to partial hepatectomy depending on pathology. Currently, surgical resection is the only therapy that is considered curative for standard gallbladder cancer, although the extent of resection is still a matter of debate. For locally advanced gallbladder cancer, neoadjuvant chemotherapy could transform primary non-resectable patients into eligible patients for resection. However, the current evidence does not cover cases of gallbladder cancer within a duplicated gallbladder [17]. More future studies and data collection would be useful to determine the most appropriate management of this rare presentation.

## Conclusions

Duplication of the gallbladder is considered a rare congenital abnormality, and the diagnosis and management of such an anomaly remain a challenge for surgeons. It remains unclear whether a simple cholecystectomy of duplicated gallbladder is sufficient versus more extensive hepatic resection for incidentally found adenocarcinoma with regard to survival of these patients.
